# GamerFit-ASD beta test: adapting an evidence-based exergaming and telehealth coaching intervention for autistic youth

**DOI:** 10.3389/fped.2023.1198000

**Published:** 2023-09-05

**Authors:** Daniel P. Hatfield, Aviva Must, Winston Kennedy, Amanda E. Staiano, James Slavet, Rachael A. Sabelli, Carol Curtin, Linda G. Bandini, Phillip Nauta, Christopher Stuetzle, April B. Bowling

**Affiliations:** ^1^ Friedman School of Nutrition Science and Policy, Tufts University, Boston, MA, United States; ^2^ Department of Public Health and Community Medicine, Tufts University, Boston, MA, United States; ^3^ Department of Nutrition and Public Health, School of Nursing and Health Sciences, Merrimack College, North Andover, MA, United States; ^4^ Eunice Kennedy Shriver Center, University of Massachusetts Chan Medical School, Worcester, MA, United States; ^5^ Pennington Biomedical Research Center, Baton Rouge, LA, United States; ^6^ Marblehead Family Therapy and Wellness, Marblehead, MA, United States; ^7^ Department of Computer and Data Sciences, School of Science and Engineering, Merrimack College, North Andover, MA, United States; ^8^ Department of Psychiatry, University of Massachusetts Chan Medical School, Worcester, MA, United States

**Keywords:** exergaming, exercise, telehealth, autism spectrum disorder, physical activity, health promotion

## Abstract

**Background:**

Health disparities faced by autistic youth are exacerbated by inadequate physical activity (PA) and sleep, whereas healthy PA and sleep may improve mood and function. Adaptive Game Squad (AGS) is an evidence-based telehealth coaching and exergaming intervention to improve PA and sleep for adolescents with diverse neurodevelopmental and psychiatric conditions. This study aimed to adapt AGS for autistic youth ages 10–15 years; beta-test the modified intervention for feasibility, accessibility, and engagement; and further refine the intervention for a larger planned demonstration pilot.

**Methods:**

Interdisciplinary experts adapted AGS to create GamerFit-ASD, a 12-week intervention that included a progressive exergame schedule, Fitbit step-tracking, weekly health coaching, and health tip/exercise videos. For beta testing, the intervention was shortened to a 4-week trial with 5 parent/child dyads. Children completed exit surveys and parents and children were interviewed about intervention feasibility, accessibility, and engagement. Exit survey data were summarized with descriptive statistics. Qualitative data were analyzed using a modified grounded-theory approach.

**Results:**

All participants (*n* = 5; ages 10–14 years) attended all 4 planned coaching sessions and completed an average of 9 of 12 planned exergame challenges for a weekly average of 50 min. All participants reported enjoying coaching sessions, 4 of 5 reported enjoying exergames, and 3 of 5 reported enjoying on-demand exercise videos. In interviews, children generally reported finding participation feasible, exergaming challenges active and fun, and coaches friendly and helpful. Parents reported high feasibility of supporting their children's involvement and valued child goal-setting and intervention flexibility; however, some found telehealth sessions overly scripted. Several adaptations to coaching scripts, coach training, and parent materials were made for the larger demonstration pilot, including changes to reduce scriptedness of coaching sessions, to provide parents with more information specific to autism, and to make video content more appropriate to children's needs/preferences.

**Discussion:**

A telehealth coaching and exergaming intervention appears feasible, accessible, and engaging for autistic youth aged 10–15. Future studies with larger, more diverse samples, longer study durations and/or follow-up periods, and more rigorous study designs are needed to advance understanding of the appropriateness and effectiveness of this type of intervention for this population.

## 1. Introduction

Approximately 1 in 36 children in the United States has autism spectrum disorder (1), a developmental disability characterized by restricted interests, repetitive behaviors, and challenges with social communication (2). Autistic children and adolescents face considerable health disparities (3–5). For example, autistic youth have about twice the risk of obesity compared to youth without autism (6). Elevated chronic disease risk in this population is partially rooted in a variety of early and persistent behavioral patterns (7, 8), including lower physical activity (PA) levels (9), poor sleep quality (10, 11), and elevated screen time (6) compared to typically developing peers. Such behaviors not only confer increased health risks across the life course but also intensify the cognitive and behavioral challenges experienced by this population (10, 12, 13). By contrast, independent of the positive cardiometabolic effects of PA, over 25 studies in individuals with ASD (14, 15) have documented associations between increased PA and improvements in anxiety (16), executive functions such as the ability to focus and self-regulate (17), and meta-cognitive processes (15). Importantly, there is emerging evidence that engaging in low to moderate PA is associated with these improvements (18, 19), increasing the viability of PA as a potential therapeutic modality.

Increased PA can also support improved sleep in autistic youth (12). This combination may be powerful, given mounting evidence that interventions increasing PA and sleep quality can produce clinically meaningful improvements in daily function among individuals with autism (10, 12, 20). In the case of PA, our team's cybercycling intervention in youth with heterogenous psychiatric and neurodevelopment disorders, including autism, reduced the odds of poor self-regulation and disciplinary time out of class by 32%–51% (21). Likewise, for sleep, a randomized controlled PA trial conducted among autistic youth found that significant improvements in sleep predicted improved inhibitory control (12). Despite the potential benefits of increasing both PA and sleep in autistic youth, it is rare for behavior change interventions to address multiple needs by targeting both behaviors.

Autistic youth face significant barriers to PA and planned exercise engagement (9, 22). A recent systematic review by our team (23) found key structural, caregiver, and child barriers to participation in physical education classes (including adaptive classes) and in community-based PA programming and exercise interventions. Most exercise interventions are delivered face-to-face and target younger autistic children, rather than those in the pre-teen and teen years, when unhealthy habits tend to increase (24, 25). A common focus on high-intensity exercise in group environments, which are often loud and chaotic, discourages participation and retention among many autistic youth (26, 27). Moreover, focusing on in-person interventions is resource-intensive and depends on often depleted familial social capital (22).

Exergames are video games that require physical movement to play. They are relatively inexpensive, require little space to play, can be played alone or with a partner, and can be targeted for a variety of developmental levels. Because they explicitly meet the preferences of young people, exergames have been used effectively to improve moderate-to-vigorous PA (MVPA) in autistic teens (28). Additionally, telehealth coaching has also been used effectively to deliver behavioral health interventions to autistic youth and their families, lowering intervention resource intensity and reducing barriers to engagement (29). However, no prior interventions have integrated exergaming with telehealth coaching to promote PA engagement and improve sleep among autistic youth.

The present study aimed to fill this gap by modifying Adaptive GameSquad (AGS), an exergaming and telehealth coaching program, specifically for autistic children. Prior to the present study, the AGS intervention was developed by adapting GameSquad, a Social Cognitive Theory-based exergaming and telehealth coaching program that improved MVPA and BMI in racially diverse children ages 10–12 years with overweight and obesity (30). This adaptation was informed by the disability-health Empowerment Model (31), and AGS's feasibility was tested for use among older youth (12–17 years) with heterogeneous neurodevelopmental and psychiatric diagnoses, including autism (32). Although the feasibility trial indicated that AGS was appealing and engaging for younger autistic participants, it was not specifically developed for autistic youth. It was also not designed to be appropriate for those with a co-occurring intellectual disability, which affects about 38% of autistic children (1). In addition, the games were not tested for useability for participants who were younger than 12, and the AGS coaching scripts were originally developed for use with older teens. To our knowledge, no prior studies have modified a telehealth coaching and exergaming intervention specifically for autistic pre-teens and teens or tested such an intervention for feasibility, acceptability, and accessibility.

To address that knowledge gap, the current study aimed to (1) use an inclusion team science approach (33) to modify the original AGS intervention and materials specifically for youth ages 10–15 years with ASD; (2) beta-test the modified intervention for feasibility, accessibility, and engagement; and (3) use beta-testing results to further refine the intervention for feasibility and preliminary efficacy testing in a larger 12-week demonstration pilot.

## 2. Methods and materials

**Figure 1:**
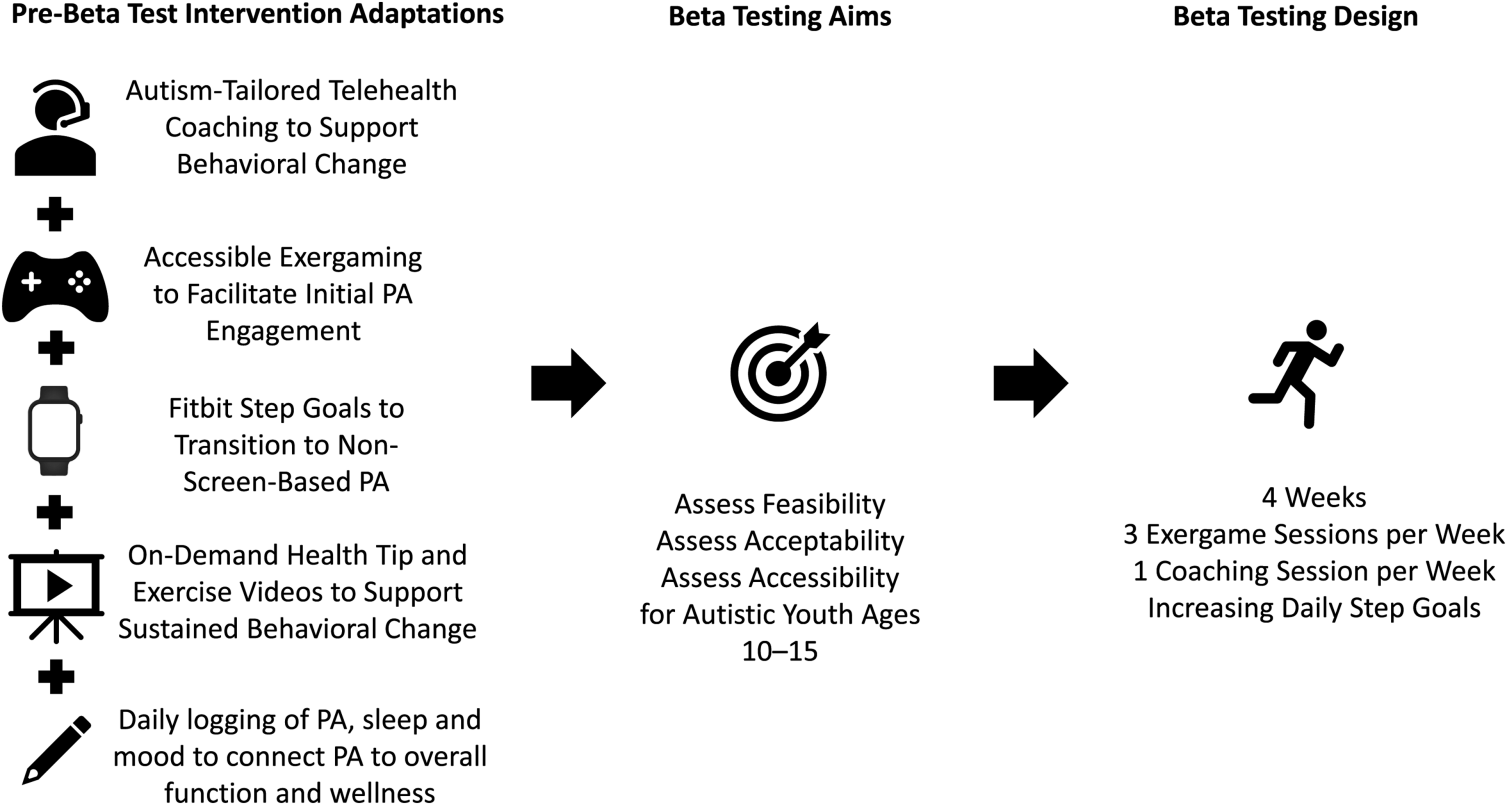
Conceptual framework for the GamerFit-ASD beta test.

Figure 1 depicts the conceptual model of this study, including the initial adaptation of the AGS intervention, the aims of the beta test, and the beta test design, and the sections that follow describe how this model was operationalized.

All study procedures and materials were approved by the Tufts University Institutional Review Board (IRB).

### 2.1. Pre-beta test intervention adaptation

The original AGS intervention was a 10-week intervention featuring a paper-based challenge menu provided to participants along with necessary equipment (Xbox, three exergames, and Fitbit). The intervention is extensively described elsewhere in the literature (34), so only a summary overview relevant to the modifications undertaken is provided here.

In the original AGS, an Xbox was installed in each participant's home by study staff, and participants played a specific selection of exergames, presented on a challenge menu, three times per week, building from 10 min to 45 min daily over the span of the intervention. Participants were also challenged to meet incrementally increasing daily step goals tracked by a Fitbit that was wirelessly uploaded to their coach. In the original AGS, the participants and a parent/caregiver met with the coach via Skype on the Xbox each week for the first 2 weeks, and then every other week for the remainder of the intervention.

To further adapt the AGS intervention, we used an inclusion team science approach, whereby health researchers who work with the general population work collaboratively with disability researchers to adapt health-promotion programs (33). To that end, the research team included an autistic adult consultant, the parent of an autistic teen, a developmental psychologist, a pediatrician, a clinical psychologist, a clinical social worker, and behavioral scientists with expertise in adaptive PA, sleep, and nutrition interventions. Several of the disability researchers were also family members of individuals with autism and/or an intellectual disability. Components of AGS that might need to be modified for autistic youth aged 10–15, including those with an intellectual disability, were identified based on the expert opinion of the individuals on this team. These included: (1) telehealth coaching session scripts and session frequency; (2) challenge menu wording, step goals, and exergaming duration; and (3) exergame and gaming console accessibility to ensure that children of younger ages and those with mild to moderate intellectual disability could understand how to start, successfully play, and progress with the exergames.

Feedback from participants and parents in semi-structured interviews following the original AGS indicated that additional intervention components would be helpful to improve engagement, reduce parental burden, and facilitate the transition from screen-based PA (exergames) to non-screen-based PA. These additional components requiring development included: (1) an integrated delivery platform to simplify intervention access for parents; (2) enhanced technology support and troubleshooting materials; (3) motivational text messages to promote participant engagement between coaching sessions; (4) health tip videos to reinforce health literacy content introduced during coaching sessions; and (5) on-demand exercise videos to promote non-exergame-based PA and help participants meet step goals. The team attempted to address item #1 above by developing an integrated, web-based platform on which to host intervention materials, but this could not be achieved due to budget constraints and institutional restrictions regarding security. Instead, a hybrid system was developed using REDCap™ (35) to deliver electronic versions of study materials and tracking forms in addition to providing all materials to families as paper copies.

Prior to beta-testing, the research team undertook a comprehensive review of the original AGS materials and adapted them based on priority population needs and feedback from the original pilot. Adaptation also required evaluating nine different developmentally appropriate exergames on three gaming platforms (Xbox One™, PS4™, and Nintendo Switch™) and selecting both a platform and suite of games based on their suitability, accessibility, and affordability prior to beta testing. The Nintendo Switch was selected based on affordability and ease of set-up and use. Ring Fit Adventure and Just Dance 2021 were the games selected based on the variety of modalities and intensity of exercise elicited, accessibility, and perceptions of acceptability among experienced team members. Based on feedback from AGS, motivational text messages were also developed to reinforce messages from the coaching sessions (e.g., providing positive feedback for reaching goals). Finally, on-demand exercise and health tip videos were developed for beta-testing. These included four short on-demand exercise videos (two promoting aerobic activity, one promoting stretching, and one promoting simple strength exercises) and two health tip videos (one promoting healthy bedtime routines and the other promoting trying new healthy foods).

### 2.2. Beta test population

Beta-testers comprised five dyads of autistic youth ages 10–15 years and their parents. A sixth dyad, which included a child with an intellectual disability, was recruited and consented but had to withdraw before the beta test began due to unrelated medical issues. ASD and intellectual disability diagnoses were parent-reported, and dyads had to speak English or have a competent translator present for coaching sessions and interviews. Participants were recruited using clinician and social media networks, with purposive sampling to ensure representation across gender identity, age, and race/ethnicity.

### 2.3. Implementation of beta test intervention

The beta-test was a 4-week feasibility and engagement trial of the intervention with the five parent/child dyads. The original AGS intervention included professional health coaches from Pennington Biomedical Research Center (PBRC) who led all telehealth coaching sessions. In contrast, to lower costs and increase potential for future replication, the beta test used a train-the-trainer model, with two non-professional coaches taking a lead role with support from PBRC coaches. The two coaches were a health sciences graduate student and a member of the research team, neither of whom had prior telehealth coaching experience. Prior to the intervention, the coaches completed a mix of asynchronous and live training sessions. Asynchronous sessions introduced the overall study design, the exergames used in the intervention, requirements for data management and reporting, and principles of effective coaching. The trainings on coaching strategies included two modules focused on considerations for working with autistic children, one led by a research expert and the other led by an autistic adult. The live training sessions included hands-on practice with the exergames and experiential role play activities to practice using coaching scripts. This combination was consistent with research showing that optimal train-the-trainer programs use a blend of experiential activities and didactic training (36, 37).

Weekly coaching sessions were delivered via HIPAA-compliant Zoom™. Following the standardized scripts, coaches reviewed exergaming and daily step goals for the prior, current, and upcoming weeks, provided encouragement and troubleshooting support as appropriate, and discussed other health tips (e.g., for trying new foods and reducing screen use before bed). The first two sessions were delivered jointly by newly trained coaches and professional PBRC coaches; the second two sessions were led independently by the new coaches. This approach gave the new coaches the opportunity to observe and learn from the experienced coaches before leading their own sessions, mirroring the approach that was planned for the full demonstration pilot.

Prior to the intervention, participants were provided wrist-worn Fitbits (Flex 2 model), a Nintendo Switch, and the two exergames (Ring Fit Adventure and Just Dance 2021). The intervention challenge menu included daily step goals, which started at 2,000 steps per day in week 1 and increased gradually to 2,750 steps in week 4. They also included three exergaming challenges per week, including a mix of Just Dance and Ring Fit Adventure games, with the target duration of each session increasing from 10 min per session in week 1 to 30 min per session in week 4. In Just Dance games, players mirror the movements of an avatar dancing to contemporary songs and earn points for accuracy. In Ring Fit Adventure games, players battle enemies and complete missions while engaging in active movement, like running and jumping. Participants had the option of tracking steps and exergaming using a paper version of the challenge menu. They were also asked to report their progress via a REDCap™ survey at the end of each week; for each challenge they marked complete in the REDCap™ survey, they would see a celebratory gif.

Motivational text messages were sent twice weekly to the parent's phone via Twilio, a web service that integrates with REDCap™ and enables messages to be scheduled in advance for automatic delivery. Reminders to charge and sync the Fitbit and complete the challenge menu were also sent out via text twice weekly. Links to health tip videos and on-demand exercise videos, both hosted on YouTube, were sent via text in weeks 3 and 4.

### 2.4. Measures

Parents completed a demographic questionnaire after screening and consent/assent. Coaches recorded the completion of coaching sessions in REDCap™. Participants and parents reported the number of gaming sessions that were completed each week and the estimated duration of exergaming sessions via an electronic questionnaire delivered weekly via email from REDCap™. If they did not complete the weekly questionnaire, the missing information was collected during the next telehealth coaching session and entered in REDCap™. Each day parents also received a link, sent via REDCap™, for children to report their exercise behavior, sleep, and mood; these measures were used mainly for feasibility testing, not for outcome evaluation. In addition, children completed an exit survey (Supplementary Material) and children and parents completed a semi-structured interview (Supplementary Material) after the intervention concluded. The exit survey was adapted from the original AGS study and included items about whether the child enjoyed the exergames, coaching sessions, and on-demand exercise videos. The interview was aimed at understanding both participant and parent perceptions of feasibility, acceptability, and accessibility of the intervention. Interviews were approximately 45 min in duration and were conducted via Zoom™. Interviews were audio-recorded and then transcribed.

### 2.5. Data analysis

Descriptive statistics were aggregated to characterize participant demographic information. The total number of telehealth coaching sessions attended was tabulated for each participant. The number of participants indicating enjoyment of exergames, coaching sessions, and on-demand exercise videos was tabulated from the exit survey and from interview data. The Fitbit data were extracted from participants’ accounts and mean steps per day calculated across the five participants for the 4-week intervention period. Interview recordings were transcribed and themes in the three domains (feasibility, acceptability, accessibility) were coded by a primary (WK) and secondary (DH) reviewer using a modified grounded-theory approach. In this approach, themes are inductively identified from interview responses, not deductively identified according to theory (38). The primary reviewer developed an initial codebook, which was used and added to by the secondary reviewer. Thematic coding results from the primary and secondary reviewers were then reviewed for discrepancies by a third, independent reviewer (AB) and any discrepancies reviewed by the team, with tie-breaking provided by the third reviewer.

### 2.6. Post-beta test intervention refinement

Based on findings from the beta test, including participant engagement data, exit survey data, and interview results, the full intervention was further refined for the full 12-week demonstration pilot.

## 3. Results

### 3.1. Participant demographics and program involvement

Table 1 describes participant demographic information and process data. The participants were mostly male and mostly White. One participant had an intellectual disability and four did not.

**Table 1:** Participant characteristics and process data (*n* = 5).

Participant characteristics	*N* or Mean (Range)
Age in years	11.6 (10–14)
Gender	
Male	3
Female	1
Non-binary	1
Race	
White	4
African-American/Latinx^a^	1
Parent-reported intellectual disability	
Yes	1
No	4
Process data	*N* or Mean (Range)
Number of coaching sessions attended (maximum: 4)	4 (4-4)
Fitbit steps per day	8,009 (1,951–14,582)
Exergame sessions completed (maximum: 12)	9 (7–12)
Mean exergame minutes per week	50 (31–63)
Reported enjoying coaching sessions	5
Reported enjoying exergames	4
Reported enjoying on-demand exercise videos	3

^a^ The term Latinx is used here as a gender-neutral term to describe individuals of Latin American descent (39). Gender neutrality was determined to be of *a priori* importance when reporting demographics due to the high rates of gender-queer and non-binary identification among autistic youth (40).

Overall engagement in the beta test was high, with all five participants attending all four planned coaching sessions. The average number of steps per day over the course of the intervention (8,009) exceeded the intervention step targets, which increased from 2,000 in week 1 to 2,750 in week 4. On average, participants completed three quarters of the planned exergame sessions, and they averaged a total of 200 exergaming minutes over the 4-week intervention. By comparison, the target exergaming times across the 4-week intervention totaled 225 min. The majority of participants reported enjoying the coaching sessions, the exergames, and the on-demand exercise videos.

### 3.2. Intervention feasibility, acceptability, and accessibility

A number of themes emerged through the child and parent interviews. These findings are presented in the following sections, organized into sub-sections for themes related to feasibility, acceptability, and accessibility of the GamerFit-ASD intervention.

#### 3.2.1. Feasibility

Participating children generally reported being able to complete the weekly exergaming challenges, with some noting that they made changes to those challenges based on personal game preferences.

“I beat every single thing. The last week, I didn't do the Just Dance, instead I did a ton of Ring Fit, half an hour to an hour of Ring Fit instead of half an hour of Just Dance.” *(Participant 5)*

Notably, when participants were asked to reflect on what activities they would have done prior to the intervention during the time they spent exergaming, most reported that the exergames displaced sedentary activities.

“Mostly probably just texting with my friends and writing music. That's pretty much it, probably.” *(Participant 1)*

Although child participants generally recognized that the challenges became harder over the four weeks of the intervention, in terms of both the difficulty of the games and gaming duration, they generally found even the hardest weeks feasible to complete.

“The final week was the hardest, but it wasn't actually too hard.” *(Participant 5)*

However, participants did note factors that made it somewhat harder to complete the exergames, including mood and competing activities.

“It was a little hard for the schedule because sometimes I might feel down, but for the most part it was okay.” *(Participant 4)*

For child participants, feasibility of wearing the wrist-worn Fitbits was mixed compared to the exergaming. However, most participants reported wearing the device at least part of the time, though for some the amount of time was limited due to physical discomfort.

“My parents would remind me, but it also just felt uncomfortable in my hands, so sometimes I would take it off.” *(Participant 1)*

Parents likewise generally reported that it was easy overall to support their children's participation in the intervention.

“It wasn't that hard. We had some challenges at times of him not wanting to do that, not wanting to work out, which is a challenge we have in general … but it really wasn't that difficult … Some days getting him to wear the Fitbit was a struggle, but for the most part he was a willing participant and it really wasn't that hard to support him.” *(Parent 1)*

However, there were some exceptions, and one parent noted challenges with setting up the Fitbit and with managing the number of tasks required for study participants, particularly early in the intervention.

“I mean it's like, ‘Don't forget to put your Fitbit on. Don't forget to charge it overnight. Don't forget to do the form. Don't forget to fill out the form when you do the exercise.’ I mean it was pretty intensive. But I would say by the end, it was much harder in the beginning and now I'm just like it's part of our routine.” *(Parent 4)*

Participants’ parents also reported challenges completing the daily logs about children's mood because the close-ended survey options often did not reflect the how the child was actually feeling.

“I had some thoughts about the questions that were asked. I was like, ‘where's tired in the descriptor of how I feel?’… I was like, ‘what, how'd they pick these random words?’… It should be a write-in too.” *(Parent 4)*

#### 3.2.2. Acceptability

Participants generally reported enjoying the Ring Fit games. Some noted the particular appeal of the gamified elements of Ring Fit, like accumulating points.

“I enjoyed Ring Fit … I enjoyed the fact that it made games, basically.” *(Participant 1)*

However, one participant found the Ring Fit games excessively stimulating, both visually and auditorily.

“Some of the [Ring Fit] games just did not work at all, the bright colored ones with flashing lights and visual noise all over the place, which was a lot of them.” *(Participant 2)*

Compared with Ring Fit, children's enjoyment of Just Dance games was mixed. While some reported enjoying the games (or even preferring them to Ring Fit games), others did not, and lack of enjoyment was often related to disliking the songs:

“I just dreaded doing it and I hated it while I was doing it. I don't know. I really don't know why… the ones I hated the most were the novelty songs, but we didn't always have to do those.” *(Participant 1)*

Although participants generally enjoyed the games, some noted that they would value having additional options and variety in the games available.

“I think adding one more to a bit of diversity, so you're not flipping, flipping, flipping, flipping between two games. Adding one or two more would just suffice … Variety is the spice of life.” *(Participant 5)*

Notably, the one Latinx parent in the study spoke to the potential value in ensuring that games include culturally appropriate content, particularly in terms of music:

“Maybe, because I'm a Spanish girl, maybe [include] more [culturally appropriate] music.” *(Parent 3)*

Some participants indicated that even after the intervention's completion they intended to continue playing the exergames, particularly the ones that they preferred. Participants also reported high levels of engagement with monitoring their step counts through the Fitbit app. Similarly, parents reported that the aspects of the intervention that worked best for their children included having the opportunity to pursue challenges and to self-monitor progress toward goals, which kept them engaged and motivated.

“Checking his steps motivated him, he's motivated by numbers, and just the whole thing really worked. I think having the Fitbit part of it really helped him because having that, how many steps did I get today? That was something he did like to know … Because he really enjoyed getting his average up.” *(Parent 5)*

Participants also reported liking their telehealth coaches overall and enjoying the weekly coaching sessions, noting that the coaches were friendly, helpful, and easy to understand. Parents also generally had positive perceptions of coaches overall, including perceptions that the coaches were friendly, nice, and adaptable.

“I think they both were very friendly and approachable, and [coach] asked some questions when he would notice that maybe [child] hadn't worn the Fitbit and would ask him, ‘Can you tell me why? Is it uncomfortable?’ So he would engage with him in that sense. So yeah, they were both friendly and nice and I think they worked well with him.” *(Parent 1)*

On the other hand, the most common shortcoming parents reported about the coaching sessions was that at times it seemed apparent that the coaches were following a script, which may have made the sessions less engaging.

“Definitely, it felt like they were following a script and a template mostly too.” *(Parent 2)*

Parents also noted that they used both the paper log and the electronic log, accessed via a QR code, to track progress on the challenges. In some cases, those logs were used for different purposes; for example, some parents used the paper log to track activity throughout the week and then used the QR code to report overall results at the end of the week.

“I did both. I would keep track of everything [on paper] and then I would go in, scan the QR code, and do it online.” *(Parent 1)*

When asked if they would recommend participation in the intervention to other families, parents reported a positive disposition toward doing so, citing their overall positive impressions of the intervention. In particular, some parents noted that the program provided a valuable, enjoyable option for children who might typically resist PA.

“I think especially for a kid like [child] though, he wanted to exercise, but he doesn't know how to bring himself to do it. He doesn't know how to get past some of his own anxieties. So this was great for him because it helped him get past his own fears.” *(Parent 5)*

Some parents noted that their children demonstrated improvements in mood and/or in health behaviors other than PA, like improved sleep, which they attributed to participation in the intervention.

“Mood improved … He slept better. He went to sleep easier. They noticed that at school, he was just a little bit calmer…Before he would have trouble going to sleep, and when he started this, he was tired finally for the first time in a while on a regular basis, which he used to be when he was swimming regularly.” *(Parent 5)*

#### 3.2.3. Accessibility

Overall, participants found both Ring Fit and Just Dance games relatively easy to learn. In some cases, prior experience or involvement of friends or siblings helped to increase their confidence in understanding how to play the games.

“It [Ring Fit] was pretty easy. Yeah, it was really quick … I've already played Just Dance previously with some of my friends, so I already [knew how to play]”*(Participant 1)*

Despite the overall ease of learning to play the games, participants generally reported that both Ring Fit and Just Dance games did force make them to exert themselves physically, with most participants characterizing the games as eliciting at least moderate levels of exertion.

“I think it [Ring Fit] was usually a moderate workout. It wasn't that hard, but it also wasn't easy. I was usually sweating and breathing relatively heavily.” *(Participant 1)*

Participants generally reported that their gaming consoles were located in shared family spaces, like the living room, and some participants reported playing the games with siblings or parents in those spaces. Parents also noted the importance of customization and flexibility with both the Fitbit and exergaming goals. As the intervention progressed, participants tended to modify the exergaming challenges so they could play the games they liked best:

“And then the coaches gave him the permission to deviate. So at the beginning he was like, ‘Okay, I'm going to do exactly what it says.’ But then he was getting tired of Just Dance because he didn't like it. So when they were like, ‘Oh, if you want to do two Ring Fits and no Just Dance, that's okay.’ And then he was like, ‘Okay, this is great. I'm going to do that instead.’” *(Parent 5)*

Some parents also noted that it would have been useful to have even more flexibility in how children participated in the intervention, such as being more explicit about the option to swap out different exergames or providing a choice of both in-person and remote options for coaching sessions.

“I would say having options for in-person or remote. That part was important … Something that might be cool is if … there were four video game options and you pick two?” *(Parent 4)*

In terms of the aspects of the intervention that worked best for them, parents reported that information was generally easy to access and that reminder messages helped them stay on top of study tasks.

“The texts were good because that just pops up in my face and I just have to click on a thing and it goes right to that thing, and it doesn't require me finding the right device and getting the right … It doesn't require navigating a bunch of stuff. It just requires me to respond.” *(Parent 2)*

Conversely, some parents noted that the text messages, health tip videos, and on-demand exercise videos sent to parents’ mobile phones between sessions did not reach some children, and others noted that the videos were not entirely appropriate to their children's needs.

“I see that one [on-demand video]. But when I say to him, he no pay attention a lot, because [it’s] boring. That’s for adult, but not for child.” *(Parent 3)*

### 3.3. Program refinements

Given the generally positive feedback from both children and parents, most elements of the intervention were retained for delivery in a larger (*n* = 23 dyads), 12-week demonstration project. However, based on constructive feedback, several additional refinements were made to the participant orientation, videos, participant packet, coach training, coaching scripts, and evaluation forms. Table 2 outlines these modifications and the underlying rationale for each. Broadly speaking, those modifications, together with the initial intervention adaptations, were developed to be consistent with principles from the Pediatric **P**hysical **A**ctivity Engagement for **I**nvisible Social, Emotional, and Behavioral **D**isabilities (PAID) Framework (41). PAID integrates disability-specific health behavior change theoretical constructs and implementation frameworks to inform the design and evaluation of interventions aiming to improve PA engagement and other health behaviors among youth with social, emotional and behavioral disabilities (41). Table 2 additionally maps program modifications to specific constructs of the PAID Framework.

**Table 2:** Adaptations made to GamerFit-ASD.

Program component	Description of modification	Rationale	Applicable PAID framework construct
Program orientation	Original approach: participants moved immediately into completing exergames and step challenges in week 1 of the intervention.	To strengthen coach-participant relationship building and alleviate early-intervention technical challenges that some families reported in the beta test.	Parent-level facilitator (ease of use)
	Modified approach: week 1 was changed to an orientation week, focused on building rapport with the child and parent, explaining what participants could expect each week, and providing technical assistance on set-up and utilization of the Fitbit, Switch console, and exergames.		
Videos	Original approach: did not offer health tip videos or on-demand exercise videos.	To facilitate non-screen-based physical activity (PA) of different types (stretching, strength training, interval training) and to reinforce live health coaching with accessible health literacy videos.	Child-level self-efficacy (goal setting, action planning); reinforcement management and stimulus control (exploration of preferred PA, choice of PA modality and intensity); environmental facilitators (ease of participation, sensory aspects of programming setting)
	Modified approach: new health tip and on-demand exercise videos were created with detailed instructions and peer modeling.		
Participant packet	Original approach: a participant packet provided to children and families included information about the intervention.	To reinforce for parents the importance of healthy habits specifically for autistic youth and to clarify the rationale for the health tip and exercise videos, with the goal of encouraging parents to share them with their children.	Parent-level outcome expectations (explicitly connect PA to child symptom alleviation and overall wellness)
	Modified approach: the packet was updated to provide information on new topics including how GamerFit-ASD was tailored for autistic participants and specific considerations and tips related to PA, sleep, screentime reduction, and healthy eating for autistic youth.		
Coaching scripts	Original approach: scripts for weekly coaching sessions included word-for-word language that coaches could use to cover key topics. These scripts were originally developed for older teens with heterogeneous mental health and neurodevelopmental conditions.	To tailor coaching for younger autistic participants who may also have mild to moderate intellectual disabilities and to mitigate issues with scriptedness of coaching sessions noted by parents during the beta test.	Child-level outcome expectations (connect PA to symptom alleviation and overall wellness); self-efficacy (individualized goal setting, troubleshooting, and action planning)
	Modified approach: scripts were modified to describe topic areas in accessible terms but not to provide word-for-word scripting. Scripts were also expanded to include reviews of on-demand exercise videos and health tip videos, as well as to discuss participant PA, sleep, and mood logs.		
Coach training	Original approach: the first two telehealth sessions were led jointly with professional Pennington Biomedical Research Center coaches.	To give coaches extra time to build confidence and comfort delivering the sessions in a non-scripted way.	Interventionist-level support and training
	Modified approach: the number of sessions conducted jointly with PBRC coaches was extended to three.		
Forms for reporting mood	Original approach: no daily log was provided to connect PA, sleep, and mood.	To address parent-reported issues with the close-ended options not always including descriptors that represented their child's actual mood.	Child-level outcome expectations (connect PA to symptom alleviation and overall wellness); reinforcement management and stimulus control (participatory design)
	Modified approach: daily log was added. After beta testing, the mood item in the daily log was modified to include an “other” option with an open-ended write-in field.		

## 4. Discussion

Although telehealth coaching and exergaming have shown promise in typically developing youth (34, 42), the feasibility and acceptability of these strategies in autistic youth is less well understood. This study aimed to address this knowledge gap by further adapting a previously tested telehealth coaching and exergaming intervention (32) for youth aged 10–15 with autism and testing the intervention through a 4-week beta test. This test demonstrated good feasibility, with all child-parent pairs participating in all four telehealth coaching sessions and children completing three-quarters of the planned exergaming sessions on average. Findings from qualitative interviews also demonstrated high acceptability and accessibility of the intervention for participating children and parents. Overall, participating children, including the one child with parent-reported intellectual disability, reported finding the exergames easy to learn and the challenges feasible to complete, even as the games increased in intensity and duration over the four-week intervention period. They also reported that the games generally led to at least moderate-intensity exertion and most noted that the games displaced sedentary activities. These findings provide encouraging evidence that the intervention has the potential to contribute additively to daily MVPA, though additional evidence is needed to confirm this outcome.

The engagement levels observed in this study are slightly higher than those observed in the original AGS study (32), in which participants completed most coaching sessions (5 of 6 on average) and completed an average of 17 of the 30 planned exergaming sessions (57%). Anecdotally, our study team observed that children with ASD in that study (*n* = 5 of 11 total participants in the intervention group, all aged 12 or older, and without ID) demonstrated particularly high levels of engagement. The current study provides further evidence of the feasibility of this approach for autistic youth aged 10–15.

Parents’ overall positive feedback regarding the intervention's feasibility and exergaming's possible impact on their children's behavior is encouraging given the importance of parental support for children's PA (43). Parents’ perceptions that it was easy to support their children's participation is especially encouraging given the depleted reserve capacity that can result from the demands associated with managing a child's disability (22).

Some parents noted that the intervention was particularly acceptable and effective for children who might not typically be drawn to other PA opportunities. This finding is consistent with prior studies that have found high acceptability of exergames like Just Dance for youth with autism (38). Finding non-traditional opportunities for PA may be particularly impactful for autistic youth, who often resist participation in traditional group PA programs, which tend to be loud, chaotic, and focused on high-intensity PA (26, 27).

One important success factor for the intervention was the telehealth coaches, whom both participants and parents characterized as fun, friendly, and easy to understand. Coaches played a particularly key role in helping the participants customize exergaming challenges and step goals to ensure they were appropriate to individual abilities and interests. In future exergaming interventions for autistic youth, this type of individual-level tailoring may likewise be important to participant engagement.

This study has several limitations that are important to note. The sample size of the beta test was small; however, it was appropriate to our study purpose and yielded useful insights about feasibility and engagement. A larger demonstration pilot is currently underway using the intervention refinements made from the beta test. In addition, while the intervention was adapted to be appropriate for autistic children with or without intellectual disabilities, only one participant in the study had an intellectual disability, making it difficult to evaluate the intervention's feasibility, acceptability, and accessibility for this subgroup. The full demonstration pilot will provide opportunities to explore these questions. Additionally, the beta test was also only 4 weeks in duration, and, although several participants noted plans to continue playing the exergames, it is unclear the extent to which engagement was maintained beyond the intervention period. Notably, in the 10-week AGS intervention, completion of the weekly exergaming sessions and total exergaming duration decreased among participants after weeks 7/8 of the program (32), so it is plausible that in this study engagement would have fallen off had the beta test been longer. Studies with longer intervention periods and post-intervention follow-up assessments are needed and, as previously noted, are currently underway. It is also possible that additional variety in gaming options may be necessary to sustain engagement over time, given that both children and parents noted the potential benefits of such variety. This will necessitate ongoing accessibility testing of new games that are always emerging on the market. Finally, future studies should also include larger, more diverse samples and more rigorous study designs (e.g., randomized controlled trials) to advance understanding of the effectiveness of this type of intervention for different communities and cultural contexts.

Overall, this study provides encouraging evidence that telehealth coaching and exergaming interventions, which have shown promise in typically developing children and older children with heterogeneous developmental and psychiatric disorders, may also be feasible and acceptable for autistic children aged 10–15. If shown to be effective in improving both PA and sleep in this population, approaches like GamerFit-ASD could be integrated into clinical treatment and school supports to improve both behavioral function and physical health in autistic youth.

## Data Availability

The raw data supporting the conclusions of this article will be made available by the authors, without undue reservation.
