# Supplementary material for: GamerFit-ASD beta test: adapting an evidence-based exergaming and telehealth coaching intervention for autistic youth
Source: Front Pediatr. 2023 Sep 5;11:1198000. doi: 10.3389/fped.2023.1198000 (PMC10507699; doi:10.3389/fped.2023.1198000)
Supplement: Supplementary file 1 [file Datasheet1.pdf]

Participant ID#___________________________________

Interviewer_______________________________________

Date Administered________________________________

Interviewer instructions: Parents can help participants answer questions as necessary. Make sure you utilize prompts to get complete answers.

Interviewer Script (in bold throughout):

Hello (participant and parent)! Thank you being beta testers for us. As you know, the purpose of a beta test is to get feedback to improve things, so please don't hold back.

This interview should take about 40 minutes total). The first set of questions are for you (the participant). These questions are about the exergames you played. The exergames are the active video games you played, Ringfit and Just Dance.

1. Were you able to play the GamerFit exergames as outlined in your exergame challenge menu? (show the menu)

A) If not, why?

B) If yes, were you able to play every week of the program?

a. Were some weeks harder than others? If so, why?

2. Was it hard to play the GamerFit exergames at least three times per week?

A) If you said it was hard, what were the reasons?

3. Did you enjoy playing RingFit?

A) If not, why?

B) If so, what made it fun?

4. Did you enjoy playing Just Dance?

A) If not, why?

B) If so, what made it fun?

5. How hard was it to learn how to play RingFit?

6. How hard was it to learn how to play Just Dance?

7. How hard did you usually feel like you were working when you played RingFit?

8. How hard di you usually feel like you were working when you played Just Dance?

9. Which game did you like better, RingFit or Just Dance?

10. Did we give you enough games to play?

A) Did you get bored with either game?

11. Did you play the GamerFit games with other people?

A) If so, who did you play with?

12. Where was your gaming console located most of the time?

13. Can you think back to the time when you did not have the GamerFit games yet, what did you do with the time you now spend playing the active GamerFit games?

14. Do you want to keep playing RingFit? Just Dance?

15. Do you think you will keep playing RingFit? Just Dance?

Now I'm going to ask you about some other parts of the program. First, I'm going to ask you about the Fitbit you wore during the four weeks.

16. How much did you wear the Fitbit during the four weeks?

17. How much did the Fitbit help you to monitor your steps?

18. How much did you like wearing the Fitbit?

19. How often did you use the Fitbit app?

Now I'm going to ask you some questions about your coaching sessions.

20. What did you think about the coaching sessions?

21. Was your coach friendly enough?

22. Was your coach easy to understand?

23. Did your coach give you healthy living tips you could use?

24. What would you change about the coaching sessions?

25. Did the motivational text messages your coach sent help you stick with the program?
A. If not, why? Were some better than others?

B. What would you change about the text messages?

Now I'm going to ask you about the on-demand exercise videos your coach asked you to watch.

26. Did you watch any of the on-demand exercise videos?

A. If not, why?

B. If yes, did you do the exercises?

a. If no, why?

b. If yes, how hard were the exercises to do?

c. Did you enjoy the exercise videos?

d. What would you change about the exercise videos?

e. Would you use them again?

f. Were they easy to follow?

g. How was the music?

h. How clear were the instructions?

Now I'm going to ask you about the health tip videos your coach asked you to watch.

27. Did you watch either of the two health tip videos?

A. If not, why?

B. If yes, did you try out the tips?

i. If not, why?

j. If so, what happened?

k. What would you change about the health tip videos?

l. Would you use them again?

Thank you so much (participant name). Is there anything else you'd like to tell me about the program before I ask your parent a few questions?

Ok (parent name). Thank you for taking the time to answer a few questions from your perspective.

1. Overall, how difficult was it to support your child taking part in this program?
    a. How difficult was it to manage the technology in particular (gaming system, Fitbit, surveys)?
2. What aspects of the program worked well for your child and why?
3. What aspects of the program worked well for you and why?
4. What aspects of the program didn't work so well for your child?
5. What aspects of the program didn't work well for you?
6. Did you perceive any effects of exercise on your child's mood or behavior?
7. Did another parent or sibling support your child's participation?
8. Did you and your child use the QR codes to fill out the electronic version of the exergame menu or did you use the paper copy?
9. How well did the coach work with your child?
10. What other suggestions do you have for how we could improve the program?
11. Would you recommend this program to another family?
12. Do you have anything else you would like to add?

Thank you again for your time.
