# Supplementary material for: GamerFit-ASD beta test: adapting an evidence-based exergaming and telehealth coaching intervention for autistic youth
Source: Front Pediatr. 2023 Sep 5;11:1198000. doi: 10.3389/fped.2023.1198000 (PMC10507699; doi:10.3389/fped.2023.1198000)
Supplement: Supplementary file 2 [file Datasheet2.pdf]

Exit Survey

Please have your child complete the survey below. Parents may assist their children when needed.

Thank you!

---

1. Did you succeed in playing the GamerFit exergames as outlined in your challenge booklet?

- ◯ Always or almost always
- ◯ Sometimes
- ◯ Never

---

2. Was it hard to play the GamerFit exergames at least three times per week?

- ◯ Yes
- ◯ No

---

3. If you said it was hard, what were the reasons? (you can check all that apply)

- ☐ I was not allowed by my parents
- ☐ There was somebody else playing on my console
- ☐ The console wasn't working
- ☐ The games did not respond well to my movements
- ☐ The games were too tiring
- ☐ The games were too boring
- ☐ We do not have enough space to play the games well
- ☐ I did not have enough games
- ☐ I did not have enough time
- ☐ I think the games are stupid
- ☐ I had too many other things to do
- ☐ There wasn't somebody to play with and it is boring to play the games by myself
- ☐ I was injured
- ☐ I would rather play non-active games
- ☐ Other

---

4. If you said other above, please explain:

____

	Totally disagree	Kind of disagree	Neutral	Kind of agree	Totally agree
5. I enjoy playing the GamerFit games.	◯	◯	◯	◯	◯
6. Playing the GamerFit games is fun to do.	◯	◯	◯	◯	◯
7. I think playing the GamerFit games is boring.	◯	◯	◯	◯	◯
8. Playing the GamerFit games could hold my attention.	◯	◯	◯	◯	◯
9. I would describe playing the GamerFit games as very interesting.	◯	◯	◯	◯	◯
10. I think playing the GamerFit games is quite enjoyable.	◯	◯	◯	◯	◯
11. While playing the GamerFit games, I was thinking about how much I enjoyed it.	◯	◯	◯	◯	◯

12. It's easy for me to learn how the GamerFit games work.	○	○	○	○	○
13. Playing the GamerFit games is easy for me.	○	○	○	○	○
14. I believe I am good at playing GamerFit games.	○	○	○	○	○
15. I think I am better at playing the GamerFit games than other people of my age and gender.	○	○	○	○	○
16. I am generally happy with my GamerFit gaming performance.	○	○	○	○	○
17. I intend to continue playing the GamerFit games.	○	○	○	○	○
18. I enjoyed the virtual coaching sessions.	○	○	○	○	○
19. I felt the virtual coaching sessions were helpful.	○	○	○	○	○
20. My coach gave me healthy tips I could use.	○	○	○	○	○
21. The motivational text messages were helpful.	○	○	○	○	○
22. The on-demand exercise videos were helpful.	○	○	○	○	○
23. The health tip videos were helpful.	○	○	○	○	○
24. The GamerFit website was easy to use.	○	○	○	○	○

25. Which GamerFit game did you play the most?	○ RingFit<br>○ Just Dance
26. Which GamerFit game did you like the most?	○ RingFit<br>○ Just Dance
27. What GamerFit game did you like least?	○ RingFit<br>○ Just Dance
28. How would you rate the intensity of playing the GamerFit games?	○ little body movements are required while playing and my breathing rate does not speed up<br>○ I move my body quite a lot while playing and my breathing rate speeds up a little bit<br>○ I have to move a lot and fast, I get out of breath, and I am sweating while playing
29. What do you think about the number of provided games?	○ Too few<br>○ About right<br>○ Too many

30. Did you buy, receive, borrow, or download other exergames in addition to the games we provided to you?	◯ Yes<br>◯ No
31. With whom do you usually play the GamerFit games?	◯ By myself<br>◯ With my parent(s)<br>◯ With my brother/sister<br>◯ With my friend(s)<br>◯ With someone else
32. Where was your gaming console located most of the time?	◯ Living room<br>◯ My bedroom<br>◯ Parent bedroom<br>◯ Sibling bedroom<br>◯ Shared game room<br>◯ Other
33. Did you hurt or injure yourself during playing the GamerFit games?	◯ Yes<br>◯ No
34. If you hurt yourself, what kind of injury was it?	◯ Bruised something<br>◯ Scratch something<br>◯ Broke a bone<br>◯ Strained a muscle<br>◯ Other
35. How much did you wear the FitBit during the 4-weeks?	◯ Almost everyday or everyday<br>◯ Most days<br>◯ Some days<br>◯ No days
36. How much did the Fitbit help you to monitor your steps?	◯ Not at all<br>◯ Somewhat<br>◯ A lot
37. Did the texts your coach sent you help you keep motivated?	◯ Not at all<br>◯ Slightly<br>◯ Moderately<br>◯ Very<br>◯ Extremely
38. How easy was it for your child to participate in this program?	◯ Not at all easy<br>◯ Somewhat easy<br>◯ Very easy
39. Please write in any comments about the program you would like to share.	______________________________
